# SUREHYP: An Open Source Python Package for Preprocessing Hyperion Radiance Data and Retrieving Surface Reflectance

**DOI:** 10.3390/s22239205

**Published:** 2022-11-26

**Authors:** Thomas Miraglio, Nicholas C. Coops

**Affiliations:** Integrated Remote Sensing Studio, Department of Forest Resources Management, University of British Columbia, 2424 Main Mall, Vancouver, BC V6T 1Z4, Canada

**Keywords:** atmospheric correction, batch processing, satellite

## Abstract

Surface reflectance is an essential product from remote sensing Earth observations critical for a wide variety of applications, including consistent land cover mapping and change, and estimation of vegetation attributes. From 2000 to 2017 the Earth Observing-1 Hyperion instrument acquired the first satellite based hyperspectral image archive from space resulting in over 83,138 publicly available images. Hyperion imagery however requires significant preprocessing to derive surface reflectance. SUREHYP is a Python package designed to process batches of Hyperion images, bringing together a number of published algorithms and methods to correct at sensor radiance and derive surface reflectance. In this paper, we present the SUREHYP workflow and demonstrate its application on Hyperion imagery. Results indicate SUREHYP produces flat terrain surface reflectance results comparable to commercially available software, with reflectance values for the whole spectral range almost entirely within 10% of the software’s over a reference target, yet it is publicly available and open source, allowing the exploitation of this valuable hyperspectral archive on a global scale.

## 1. Introduction

Remote sensing Earth observations are used extensively for the estimation of vegetation attributes and land cover/land cover change monitoring [1,2,3,4] due to their ability to image large swathes of the Earth surface and revisit sites of interest. Images acquired by satellites equipped with multispectral sensors, such as Landsat [5], Sentinel-2A/B [6], or Terra and Aqua [7] are preprocessed to produce analysis ready data products, facilitating fast and accurate application development [8]. The first hyperspectral satellite sensor, Hyperion [9], launched on EO-1 in 2000 acquired images over 17 years, leading to a significant image archive of hyperspectral data [10]. Hyperion images are distributed as at-satellite radiance data and as a result require significant preprocessing before use, due to a poor calibration of the pushbroom detector leading to along-track striping and the presence of a spectral smile that must be addressed [11]. In order to derive surface reflectance image-based or physically-based atmospheric correction (AC) methods must then be applied.

A number of methods have been published to preprocess Hyperion data. Scheffler and Karrasch [12], for example, evaluated the performances of six destriping algorithms, either distributed as part of the ERDAS Imagine, Environment for Visualizing Images (ENVI) software, or that have to be implemented by users. More recently, Pal et al. [13] presented a new method based on local statistics to reduce striping in Hyperion images. To estimate surface reflectance, most AC methods used with Hyperion images have relied on processors using radiative transfer models (RTM), such as ATCOR [14], FLAASH [15], or other specific algorithms [16,17,18,19,20,21,22]. However, empirical or analytical methods can also be used: Prieto-Amparan et al. [23] demonstrated that the empirical line [24] could be sufficient for biomass estimation of grasslands, and Katkovsky et al. [25] proposed an algorithm based on the solutions of radiative transfer equations, and demonstrated that its results were in agreement with the FLAASH outputs when used over Hyperion images. Ientilucci and Adler-Golden [26] presented an overview of various atmospheric correction methods and software dedicated to hyperspectral imagery.

While many of these approaches have been applied to Hyperion imagery successfully, there is no definite software package available which can guide the user through the entire process of obtaining surface reflectance from the downloaded Hyperion at-sensor radiance. This lack of access to a free and open framework to bring together these published approaches limits the large-scale use of Hyperion data. Indeed, ease of access to remote sensing data is critical for their use by the scientific community, as preprocessing steps may be difficult to implement for non specialists. In the same spirit, Petropoulos and Anagnostopoulos [27] developed the freely distributed SEVIRI PrePro software to process data from the SEVIRI satellite sensor, and more recently Li et al. [28] proposed Python and Javascript tools to produce user-ready Landsat images from Google Earth Engine (GEE).

In this paper, we present SUREHYP, a Python library aggregating multiple methods to preprocess and retrieve surface reflectance from the Hyperion radiance data distributed by the USGS (earthexplorer.usgs.gov, accessed 7 February 2022). The framework utilizes GEE and the SMARTS radiative transfer model [29,30], both freely available, to perform the AC. Users can also easily adapt it to their specific needs due to the widespread use of Python in the scientific community, and suggest improvements or additional methods to complement the existing ones. The source code is freely available on GitHub (tmiraglio/SUREHYP, accessed on 19 October 2022) and the library can be installed as a pip package.

This paper presents the methods implemented into SUREHYP for the preprocessing, as well as the AC algorithm available to retrieve surface reflectance. A demonstration case is provided, SUREHYP and FLAASH are used to retrieve surface reflectance and their outputs are compared.

## 2. Materials and Methods

### 2.1. Hyperion Data

**Figure 1:**
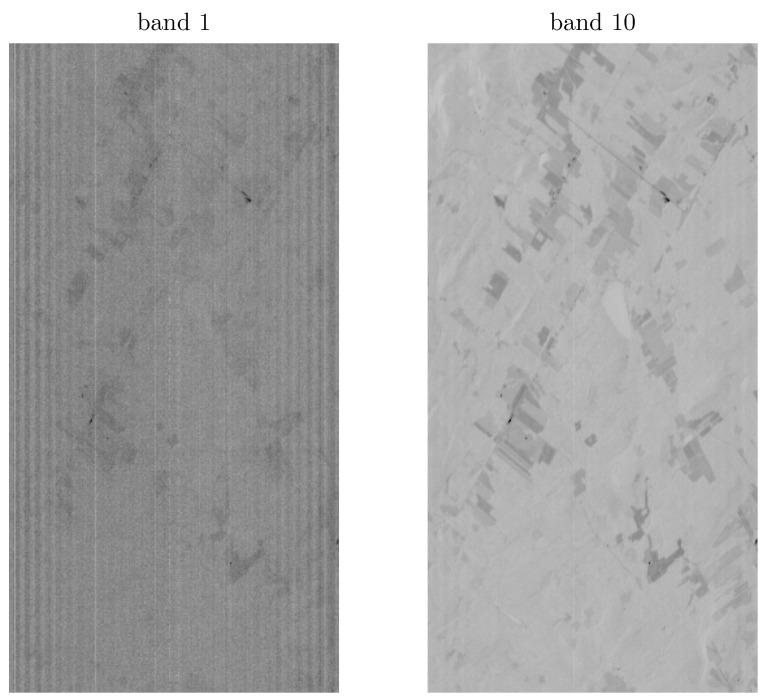
Raw hyperion bands from the L1R dataset. Although present in all bands, striping is clearly visible in band 1.

The Hyperion sensor used two spectrometers covering the visible and near-infrared (VNIR) and the shortwave-infrared (SWIR), with a four bands of overlap between the VNIR and SWIR arrays. As a result the imagery consists of 242 bands covering the 356–2577 nm range with a spectral bandwidth of 10 nm and a spatial resolution of 30 m. Hyperion data is distributed by the USGS at three different processing levels: L1R, L1Gst, and L1T. The L1R dataset is distributed as HDF4 files, and contains radiometrically-corrected images that are not terrain-corrected. Two spectral bands of a Hyperion L1R dataset are shown in Figure 1 and demonstrate a number of preprocessing challenges including the spectral smile and stripping. The L1Gst and L1T datasets are radiometrically- and terrain-corrected images and distributed as GeoTIFF files. However, due to the georeferencing, rows and columns do not correspond to track directions. Along with these datasets, informations pertaining to the acquisition dates and conditions, such as the solar and satellite angles, are available.

### 2.2. Workflow

**Figure 2:**
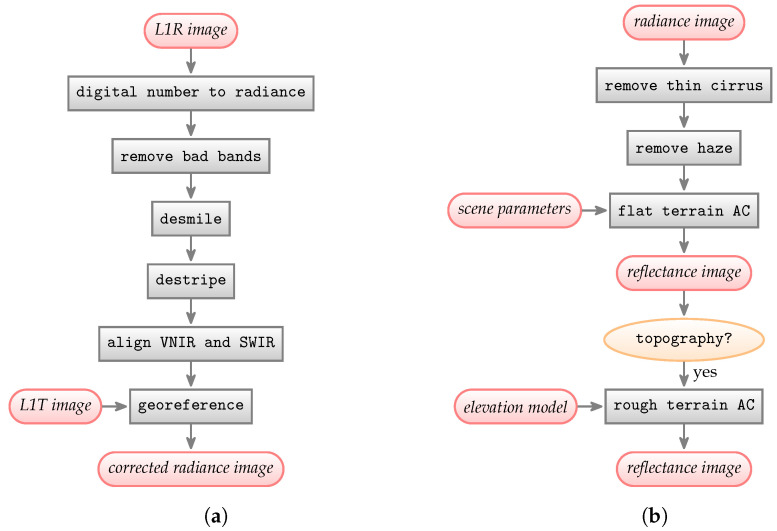
Workflow of SUREHYP to retrieve surface reflectance from Hyperion L1R data. (**a**) Preprocessing steps; (**b**) Atmospheric correction steps.

SUREHYP has two main components. First, the framework applies corrections specific to Hyperion, such as desmiling, destriping, and alignment of the VNIR and SWIR bands. The desmiling and destriping are related to the sensor across- and along-track directions, and have to be done on the L1R data. Second, once the sensor radiance is corrected, atmospheric correction can be applied to derive surface reflectance. The workflow of SUREHYP is presented in Figure 2.

### 2.3. SUREHYP Preprocessing

Hyperion images are delivered as digital numbers that are converted to radiance values by dividing VNIR bands by 40 and SWIR bands by 80. However only 200 of the 242 bands were actually calibrated, and bands 1 to 7, 58 to 76, and 225 to 242 were unused [11] and need to be removed before additional processing.

#### 2.3.1. Desmiling

**Figure 3:**
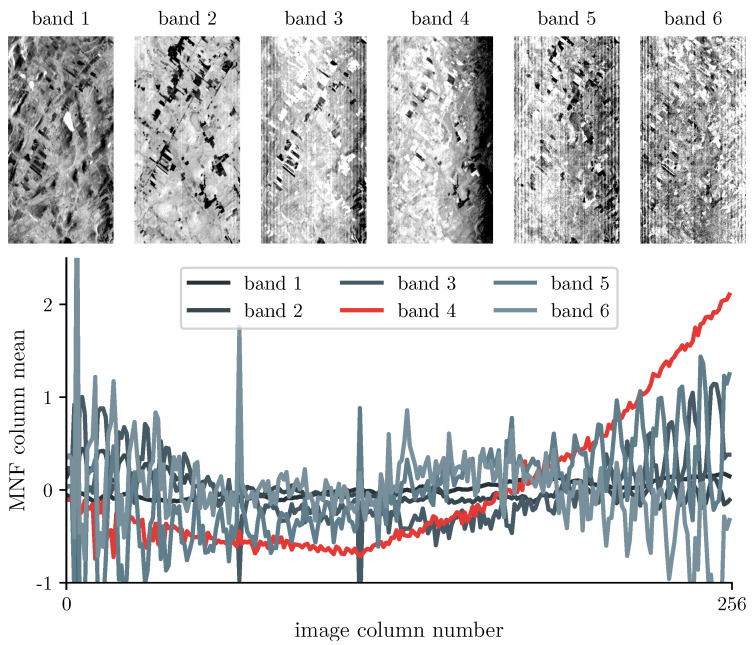
First six bands of the VNIR array in the MNF space computed using the whole Hyperion image, and associated column means. The spectral smile can be observed on band 4.

To correct the spectral smile on Hyperion bands, the across-track illumination correction presented by San and Süzen [31] is implemented. This method assumes that, after transformation in the Minimum Noise Fraction (MNF [32]) space, the spectral smile, if present, is visible in the first band of the array. To remove the smile, a polynomial function is fitted on the column means of the first MNF band, and its values are then subtracted to all the rows of the band with Equation (1):

(1) $$x_{ij}^{\prime }=x_{ij}-p_{i}$$

with $x_{ij}^{\prime }$ the desmiled MNF value, $x_{ij}$ the original MNF value, and $p_{i}$ the value of the polynom for column *i*, row *j*. The corrected MNF array is then transformed back into the radiance space. However, most of the studies using the MNF band 1 were applied over subsets of Hyperion images [1,11,31,33]; when applied over entire Hyperion images, the impact of the spectral smile may manifest on multiple MNF images (see an example in Figure 3). While not necessarily problematic when only processing single images, as the user can assess which MNF band is most affected by the spectral smile, this becomes a larger issue when processing large batches of Hyperion images as user action is unrealistic. To allow for automatic implementation, an empirical method was developed to automatically identify the smiled band: a polynomial function of degree *d* is fitted to the column means of each MNF band. Then, the polynoms for which the coefficient of order *d* is beyond three standard deviations of the mean value of all coefficients of order *d* are flagged as belonging to a smiled band, and the desmiling can proceed. The degree of the polynomial function can be user defined.

#### 2.3.2. Destriping

Two destriping methods are available in SUREHYP. The first destriping method is the local destriping presented by Datt et al. [11]: the mean value $m_{i}k$ and the standard deviation $s_{ik}$ of pixel values at column *i*, band *k* are computed. The outlier columns are detected using Equation (2):

(2) $$\frac{|m_{ik}-l_{med}\left(m_{ik}\right)|}{l_{med}\left(s_{ik}\right)}\geq \mathrm{threshold}$$

with $l_{med}$ a median filter of selectable neighbourhood along the columns, and threshold a user-defined value. The outlier pixels are then desmiled according to Equation (3):

(3) $$x_{ijk}^{\prime }=\frac{l_{mean}\left(s_{ik}\right)}{s_{ik}}x_{ijk}+l_{mean}\left(m_{ik}\right)-\frac{l_{mean}\left(s_{ik}\right)}{s_{ik}}m_{ik}$$

with $x_{ijk}^{\prime }$ the updated pixel value at row *i*, column *j*, band *k*, $l_{mean}$ a mean filter of selectable neighbourhood along the columns, and $x_{ijk}$ the original pixel values. The dimension of the selected neighbourhood and the threshold are user specified and will vary on an image-to-image basis. For instance, Datt et al. [11] used a local average of five pixels in the VNIR, and up to 41 in the SWIR, while Scheffler and Karrasch [12] used local neighbourhoods of 21 and 41 for the VNIR and SWIR, respectively. In order to remove the maximum number of stripes regardless of the width, SUREHYP iteratively lowers the neighbourhood from 21 to 5 for the VNIR and 41 to 5 for the SWIR.

The second implemented destriping method is described by Pal et al. [13] and is twofold: first, a global destriping is undergone. For each band *k* in the image, columns means $P_{ca,ik}$ are computed and the width of the largest trough/crest $n_{c}$ is measured. To do so automatically, Scipy’s find_peaks is used, and $n_{c}$ if set to the median value of the 20% largest widths, rounded up to the next integer. Then, a Savitzky-Golay filter of width 10$n_{c}$ and order 2 is applied to the columns means of each band to obtain a smoothed curve $P_{fit,ik}$. The image cleaned of global stripes is obtained with Equation (4).

(4) $$x_{ijk}^{\prime }=x_{ijk}+\left(P_{fit,ik}-P_{ca,ik}\right)$$

Finally, local stripes, that do not span entire columns of the image, can be removed. For each band, a 3$n_{c}\times$3$n_{c}$ moving window is used to compute the local mean $I_{Corr\_mean,ijk}$ and standard deviation $I_{Corr\_stdev,ijk}$. Pixels are flagged as outliers according to Equation (5):

(5) $$x_{ijk}-I_{Corr\_mean,ijk}>I_{Corr\_stdev,ijk}$$

If 90% of the pixels in a column of the moving window are flagged as outliers, then the column is flagged as a local stripe and its values replaced by those of $I_{Corr\_mean}$ for these pixels.

#### 2.3.3. Aligning VNIR and SWIR, Georeferencing

An along-track one pixel shift is present between pixels 128 and 129 of the SWIR array due to the spectrometer design. The VNIR and SWIR spectrometers were also slightly misaligned, leading to incorrect registration between VNIR and SWIR arrays [34]. The along-track shift is corrected by moving the right-hand side of the SWIR array one pixel up. To correct the registration, Khurshid et al. [34] used a counter-clockwise rotation of 0.22° of the VNIR array. Alternatively, Thenkabail et al. [35] proposed the use of the overlapping spectral bands in the VNIR and SWIR to realign the images. SUREHYP implements a mix of both methods. First, the right-hand side of the SWIR is adjusted. Then the VNIR and SWIR bands closest to 925 nm are used to detect spatial features in both images using an Oriented FAST and Rotated BRIEF (ORB) detector [36], that presents the advantage of being fast, rotation invariant, and resistant to noise. The best 15% matches are used to realign the VNIR and SWIR arrays.

The georeferencing of the corrected radiance data is similar, and uses the bands from the georeferenced L1T and the corrected L1R data corresponding to 833 nm. The processed radiance image in then saved along with its various acquisition parameters.

### 2.4. Atmospheric Correction

Numerous methods exist to implement an atmospheric correction, belonging either to the (i) image-based family (e.g., empirical line) or (ii) physically-based family (using radiative transfer processors). SUREHYP relies on a physically-based method, using the SMARTS radiative transfer model, which has been widely used [37,38,39] and is freely available at https://www.nrel.gov/grid/solar-resource/smarts.html (accessed on 19 October 2022). SMARTS requires several input variables to define the atmosphere and illumination conditions at the time of the image acquisition, including solar angles, acquisition dates, atmosphere water vapor and ozone concentrations, site altitude, or ground slope angle data. While the user can use its own inputs, SUREHYP contains submodules to retrieve these information from the Hyperion metadata, from GEE, or directly from the radiance image when possible. More specifically, the digital elevation model (DEM) and the atmosphere ozone and water vapor contents can be retrieved from GEE using datasets specified by the user. Retrieval of the water vapor concentration can also be done using water vapor absorption bands and a Look-Up Table (LUT) generated with SMARTS.

If adjacency effects are neglected, top-of-atmosphere (TOA) reflectance $\rho_{TOA}$ and the surface reflectance $\rho_{surf}$ can be derived with Equations (6) and (7) [35,40]:

(6) $$\rho_{TOA}=\frac{\pi Ld^{2}}{E_{sun}cos\left(\theta_{z}\right)}$$

(7) $$\rho_{surf}=\frac{\pi (L-L_{cirrus}-L_{haze})}{\tau_{gs}\left(\tau_{sg}E_{sun}cos\left(\theta_{z}\right)+E_{dif}+{\hat{\rho }}_{terrain}E_{g}\right)}$$

with *L* the at-satellite radiance, *d* the Earth-Sun distance correction factor, $E_{sun}$ the extraterrestrial irradiance, $\theta_{z}$ the angle of solar incidence, $L_{haze}$ the path radiance, $L_{cirrus}$ the cirrus clouds radiance, $\tau_{gs}$ the transmittance along the ground-sensor optical path, $\tau_{sg}$ the transmittance along the sun-ground optical path, $E_{dif}$ the diffuse irradiance, ${\hat{\rho }}_{terrain}$ the local terrain reflectance, and $E_{g}$ the global irradiance on the ground. $E_{g}$ is null in the case of a flat surface.

SMARTS gives $\tau_{gs}$, $\tau_{sg}$, $E_{sun}$, and the total diffuse irradiance $E_{dif}+{\hat{\rho }}_{terrain}E_{g}$, while $\theta_{z}$ is known through the acquisition parameters. The path radiance, $L_{haze}$ represents the haze radiance within the scene due to aerosols, and $L_{cirrus}$ that of transparent thin cirrus clouds. Preliminary removal of their signal from the image is necessary to perform the AC.

#### 2.4.1. Thin Cirrus and Haze Radiance Correction

Gao et al. [41] presented a thin cirrus correction method involving the 1380 nm band and more recently extended it to the 400–2450 nm range [42]. As Hyperion measures the 1380 nm band, this method was implemented in SUREHYP. From the radiance image, $\rho_{TOA}$ is computed and scatterplots of the reflectance at 1380 nm versus the reflectance at other wavelengths are used to derive a slope factor $K_{a}$ Then, the cirrus reflectance is removed using Equation (8) and the corrected $\rho_{TOA}$ is back transformed into at-satellite radiance, with $L_{cirrus}$ removed.

(8) $$x_{ijk}^{\prime }=x_{ijk}-\frac{\rho_{TOA,1380}}{K_{a,k}}$$

Haze is removed using a dark-object subtraction technique [43] on the assumption that, provided a scene is large enough, there will be a dark object within the scene whose reflectance over the entire spectrum should be close to zero, and therefore measured radiance from this object should also be close to zero. For each band, the minimum radiance value is stored. Then, as haze is due to scattering within the atmosphere, Chavez [43] argues that the relative scattering in an atmosphere follows a wavelength powerlaw $\lambda^{c}$, the *c* coefficient depending on the atmospheric conditions (−4 for a very clear atmosphere, −0.5 for a very hazy one). Therefore, the haze radiance $L_{haze}$ is obtained by fitting a $A\lambda^{c}$ function so as to be as close as possible, and below, the minimum radiance value of each band, and subtracted from the at-satellite radiance.

#### 2.4.2. Image-Based Retrieval of the Water Vapor Concentration

**Figure 4:**
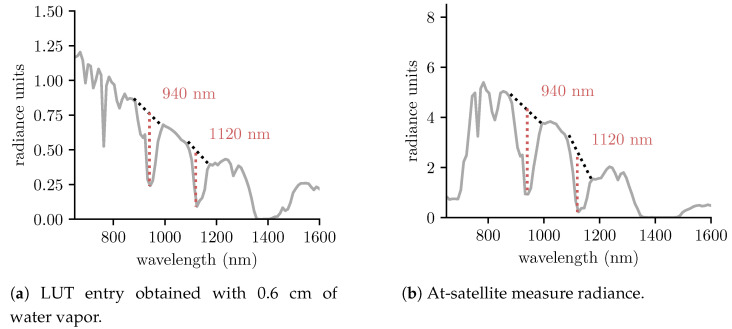
The relative absorption depths at 940 nm and 1120 nm between (**a**) the sun irradiance multiplied by the atmosphere transmittance along the sun-ground-sensor optical path, as generated by SMARTS and (**b**) at-satellite radiance are compared to estimate the water vapor concentration in the scene.

The image-based water vapor retrieval method implemented in SUREHYP relies on the water vapor absorption bands at 940 and 1120 nm, assuming that the effect of diffuse irradiance around the water absorption bands is negligible. First, a LUT is generated with SMARTS using all known scene parameters and sampling the water vapor concentration over the 0–12 cm range. The LUT links the water vapor concentration with the relative absorption depth, that is, the ratio between the mean radiance at the shoulders around the absorption band and the radiance at the absorption band (see Figure 4).

The 1120 nm Hyperion band is used by default. However, if the band is saturated, the algorithm switches to the 940 nm band to estimate water vapor content. The scene average water vapor concentration is interpolated from the LUT values for which the relative absorption depths are the closest to that of the image.

#### 2.4.3. Flat and Rough Terrain Atmospheric Corrections

Once the previous steps have been applied, the flat terrain AC can proceed using SMARTS and Equation (7). This AC assumes that the pixel terrain is a flat surface, which may be an acceptable hypothesis depending on the spatial resolution of the images and the actual terrain elevation.

If the pixel locations are heavily sloped, terrain elevation can be taken into account, requiring a DEM. SUREHYP can download a freely available DEM from GEE, or use one provided as input by the user. Terrain elevation, slopes, and slope azimuths are computed, and several SMARTS runs are used to sample the (elevation, slope, slope azimuth) space and build a LUT. The SMARTS outputs are then interpolated to obtain the irradiance values for each pixel, and surface reflectance is derived using Equation (7). However, rough terrain correction obtained with this equation, which assumes the surface to be Lambertian [44], may lead to overly bright pixels in the shadowed areas. Various methods exist to account for the bidirectional reflectance distribution function (BRDF) of the surface, Richter et al. [45] undertook a comparison and concluded that no single method performed the best, however recommended the modified Minnaert approach. From the rough surface reflectance $\rho_{rough}$, the corrected reflectance $\rho_{MM}$ is obtained using Equation (9):

(9) $$\rho_{MM}=\rho_{rough}{\left(\frac{cos(\beta )}{cos(\beta_{T})}\right)}^{b}$$

with β the local solar illumination angle, and $\beta_{T}$ and *b* set to values as per the set of rules (Equations (10) and (11)) given by Richter et al. [45]

(10) $$\beta_{T}=\left\{\begin{array}{c} \theta_{s}+20\text{°}\;\mathrm{if}\;\theta_{s}<45\text{°}\\ \theta_{s}+15\text{°}\;\mathrm{if}\;45\text{°}\leq \theta_{s}<55\text{°}\\ \theta_{s}+10\text{°}\;\mathrm{else} \end{array}\right.$$

(11) $$b=\left\{\begin{array}{c} 0.5\;\mathrm{for}\;\mathrm{non}\;\mathrm{vegetation}\\ 0.75\;\mathrm{for}\;\mathrm{vegetation}\;\mathrm{and}\;\lambda <720\;\mathrm{nm}\\ 0.33\;\mathrm{for}\;\mathrm{vegetation}\;\mathrm{and}\;\lambda \geq 720\;\mathrm{nm} \end{array}\right.$$

with $\theta_{s}$ the sun zenith angle, and λ the wavelength. Finally, the surface reflectance image is saved.

### 2.5. Reflectance Retrieval Comparison

**Figure 5:**
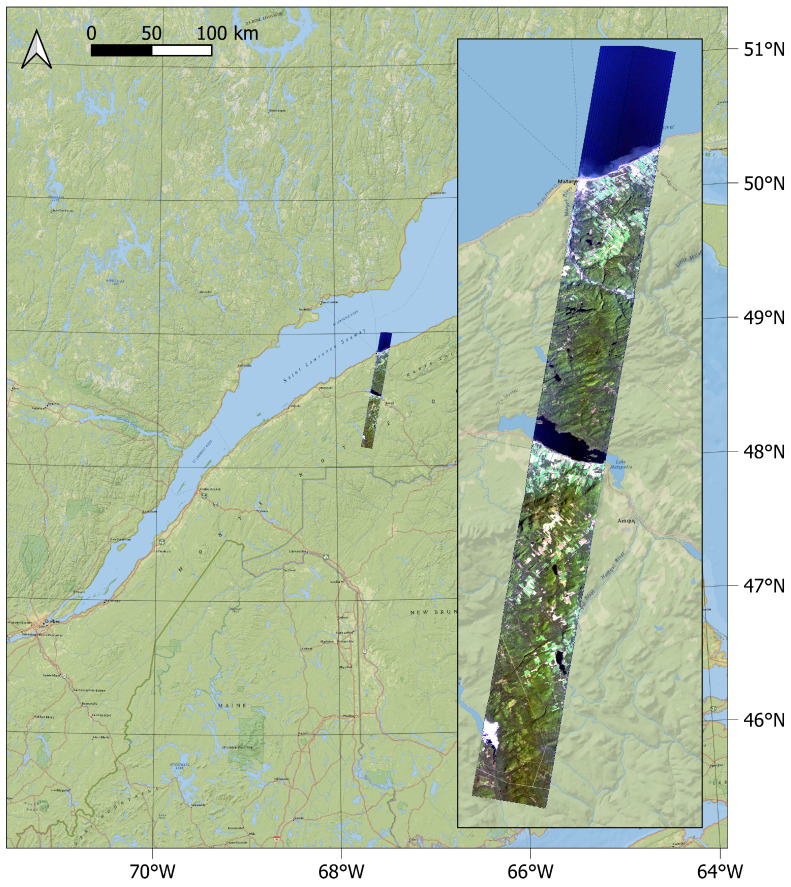
RGB (bands 29, 20, 12) composition of the Hyperion image used as a demonstration case.

As a demonstration case, SUREHYP and FLAASH are applied on Hyperion image EO1H0110262016254110KF, acquired over Québec, Canada in September 2016 (see Figure 5) at 12 h 50 GMT. This image contains water bodies (Saint Laurent River and lake Matapédia), small urbans areas, agricultural fields, forests, and has an elevation range of 0–500 m. This variety of land cover types made it a good candidate for comparison purposes, as urban areas, crop fields, and forests are all extensively studied through remote sensing. The L1R data in composed of 256 columns and 3189 rows, covering an area of 735 km${}^{2}$.

The L1R image was processed using the entire SUREHYP chain described in Figure 2. The smiled bands were detected automatically, and the destriping was done according to Pal et al. [13]. For the atmospheric correction, atmosphere was set to Sub-Arctic Summer, ozone concentration was obtained from the TOMS and OMI Merged Ozone Data using GEE, and water vapor concentration was estimated from the image using the method described in Section 2.4.2. The DEM was retrieved from the Canadian Digital Elevation Model using GEE. Both the flat terrain AC and the rough terrain AC images were saved.

FLAASH was ran with a Sub-Arctic Summer atmospheric model, water vapor concentration was retrieved using the 1120 nm absorption feature when possible. Finally, the aerosol model was set to Rural and aerosols were retrieved using the 2-Band (KT) option available. FLAASH does not consider the DEM for its atmospheric correction, and the output is therefore a flat terrain AC image.

**Figure 6:**
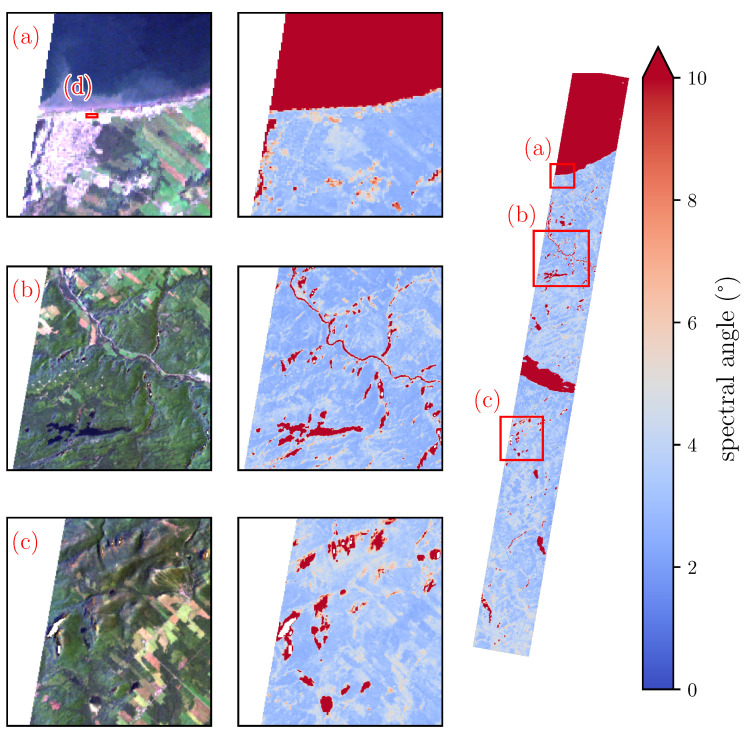
Spectral angle between the outputs of FLAASH and SUREHYP over the Hyperion image. Subscene (**a**) covers water, urban, and agricultural areas, subscene (**b**) contains agricultural areas, water, and hilly terrain, and subscene (**c**) contains hilly terrain and agricultural areas. Area (**d**) is a built-up area with a parking lot presenting a uniform reflectance of 0.2.

At the image scale, reflectance spectra produced by SUREHYP and FLAASH were compared using the spectral angle (SA) on a pixel-to-pixel basis in order to identify areas where the outputs from both software differed significantly (SA > 10°). The SA considers each spectrum as a vector in a *K*-dimensional space (with *K* the number of bands). It is obtained using Equation (12):

(12) $$\mathrm{SA}\left(f,s\right)=\arccos\left(\frac{\sum_{k=1}^{K}f_{k}s_{k}}{\sqrt{\sum_{k=1}^{K}f_{k}^{2}}\sqrt{\sum_{k=1}^{K}s_{k}^{2}}}\right)$$

with *f*, *s* the spectra that are being compared. Then, absolute and relative reflectance differences were computed over area (d) (see Figure 6), using FLAASH outputs as a reference, to assess their behaviour over the 400–2500 nm range. Area (d) is a built-up area with an almost constant reflectance value of 0.2 over the whole spectral range, which makes it easier to compare the outputs of both software at different wavelengths.

## 3. Results

### Comparison between SUREHYP and FLAASH Outputs

Figure 6 shows the spectral similarities over the whole Hyperion image. SA over the majority of landcovers was low, with values between 2 and 6° over the urban area, agricultural fields, and sunlit forests indicating high spectral similarity. Alternatively, darker objects, such as water bodies (subscenes (a) and (b)) and shadowed area (subscenes (b) and (c)) presented significant spectral dissimilarity.

**Figure 7:**
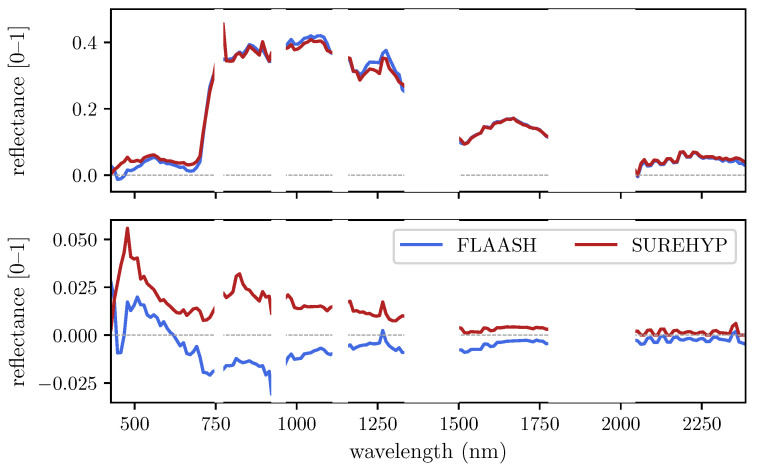
Median reflectance spectra of SUREHYP and FLAASH from (top) the areas with SA < 10° and (bottom) the areas with SA ≥ 10°.

Figure 7 shows the average reflectance spectra produced by SUREHYP and FLAASH over the high (SA < 10°) and low (SA ≥ 10°) spectral similarity areas. In the high similarity areas, it appears that SUREHYP reflectance are higher than those of FLAASH over the blue and red regions (0.04 at 475 nm and 0.02 at 675 nm at most). Higher sureface reflectance estimates are also apparent at 770 nm, just short of the oxygen absorption band. Over the green and infrared region, FLAASH and SUREHYP outputs are very similar, and are the same above 1500 nm. Around 450 nm, FLAASH produced negative reflectances.

In the low similarity areas, reflectances from both FLAASH and SUREHYP were closely matched with differences close to zero over the whole spectrum. It appeared that FLAASH almost exclusively produced negative reflectance, except in the blue and green regions. Reflectances produced by SUREHYP are all above zero, with a peak at 470 nm and a decrease until 750 nm.

**Figure 8:**
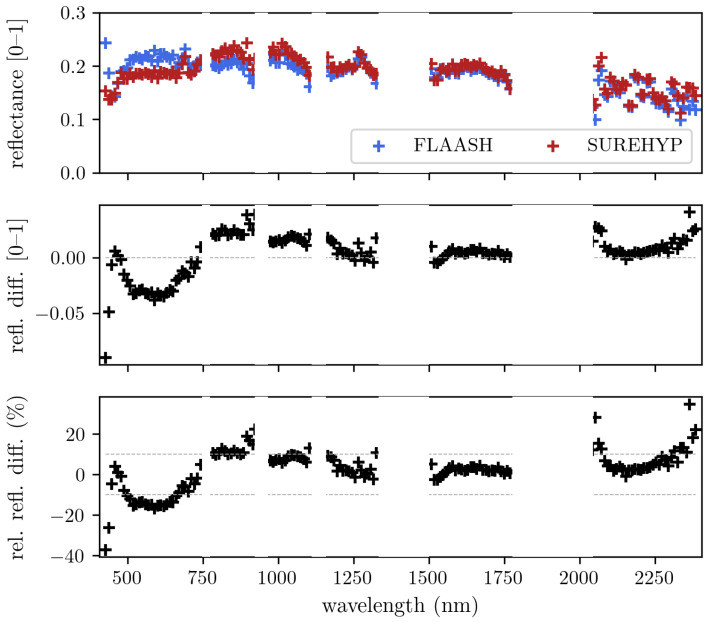
(**top**) Reflectance spectra of SUREHYP and FLAASH over area (d), (**middle**) absolute reflectance difference and (**bottom**) relative reflectance difference between SUREHYP and FLAASH outputs.

Figure 8 and Table 1 display the absolute and relative reflectance differences over area (d). Spectral differences are the most pronounced in the green region, with around 15% reflectance difference. In the NIR, SUREHYP reflectances are slightly above those of FLAASH, with values 10% higher, while for wavelengths >1000 nm, relative reflectance difference is on average 4%. Overall, over the 400–2500 nm range, SUREHYP reflectances are almost entirely within 10% of those obtained with FLAASH.

**Figure 9:**
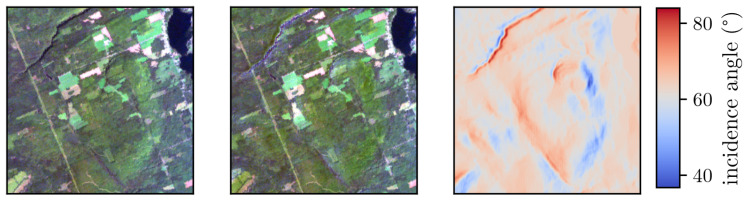
For a hilly subscene, from left to right: RGB (bands 29, 20, 12) composition of SUREHYP output with a flat AC, RGB composition of SUREHYP output with the rough terrain AC, and the solar incidence angle on the surface.

Figure 9 shows RGB compositions of the SUREHYP outputs over a hilly terrain with the flat and rough terrain AC. Over the scene, the radiation incidence angle on the surface, computed from the solar angles and the DEM, varies between 38° and 82°, while the solar zenith, which is the only angle considered for the flat terrain correction, is 63.5°. As visible in the flat AC image, not taking this variation into account leads to one side of the terrain being clearly brighter than the other. The rough terrain AC corrects for this and compensates for the variations of irradiance.

## 4. Discussion

Multiple algorithms have been proposed to preprocess Hyperion data, and various commercial software exist to perform AC and retrieve surface reflectance from the images. Previous studies have often employed FLAASH to perform AC when processing Hyperion imagery [1,20,21,22,46]. FLAASH is based on the AC model MODTRAN [47], retrieves water vapor and aerosol concentrations from the hyperspectral image, and does a flat terrain AC. While the outputs of FLAASH and other commercially available software have been shown to be accurate and satisfying for analysis purposes, this commercial distribution may limit the full exploitation of available remote sensing archives. Indeed, regardless of price, a number of the key parameters are set and not easily modified by the user. SUREHYP uses freely available software and tools (Python, SMARTS and GEE), and although the sources of the most recent SMARTS versions are not distributed, those of SMARTS v.2.9.5. are open. As it is written in Python, users are able to examine each step and easily modify the code or add additional functions of interest. Moreover, improvements and new functions can easily be implemented by the community. While SUREHYP mostly implements functions and algorithms that have been previously published, an automatic spectral smile detection algorithm was developed to facilitate batch processing of the Hyperion archive. Likewise, an algorithm to retrieve water vapor content from the images was implemented. It currently takes 68 s to preprocess a L1R Hyperion image and obtain surface reflectance using a flat terrain atmospheric correction on one core of a machine equipped with a 3.2 GHz Intel Xeon Silver 4215R CPU and 128 Gb of RAM.

Comparisons between FLAASH and SUREHYP indicate very similar outputs with the main difference occurring at shorter wavelengths, in the visible region of the spectrum (see Figure 7 and Table 1). Reflectances produced by FLAASH in the blue region were particularly low, to the point of being negative for some wavelengths <500 nm. As visible in Figure 8, in the blue, red, and SWIR regions, the median reflectance relative differences were below 10%, while in the NIR the median value was 10.7%. Overall, for wavelengths <1000 nm, relative differences were higher than for those above 1000 nm. As lower wavelengths are the most affected by aerosols and haze, it is assumed that the main cause of difference between SUREHYP and FLAASH outputs is the haze removal step, with different software estimating difference haze values. In the example image, the darkest object identified on the imagery was a shadow visible in subscene (c) of Figure 6, however haze thickness may have been different over the rest of the scene. While it may be possible to look for local dark objects and create a haze thickness map, ensuring that the pixels identified as belonging to a dark objects, and not merely to the darkest object in the area, may be difficult. Conversely, it seemed that FLAASH produced negative reflectances in the darkest areas, as well as in the blue region for the sunlit areas, which indicates over correction. However its source is unclear: as a decrease is visible between 500 and 750 nm, this may not be due to overestimated haze radiance. This overcorrection may also partially explain the differences between SUREHYP and FLAASH in the visible when performing a flat terrain AC.

The peak at 770 nm that can be observed in Figure 7 for the SUREHYP reflectances is a consequence of the oxygen absorption band. SMARTS provides the transmission and irradiance values at an interval of 1 nm over the 400–1700 nm interval, and a gaussian filtering with a 10 nm FWHM is applied to these values to match the characteristics of Hyperion. The consequence is that the 760 nm absorption feature affects the surrounding bands. As this peak should not be present, it may be that the effect of oxygen is overcorrected by SUREHYP, i.e., that the transmission at 760 nm is underestimated. Further work may therefore be necessary to fully account for the oxygen band.

## 5. Conclusions

SUREHYP is a Python package that provides several functions to preprocess Hyperion data and obtain analysis-ready surface reflectance images. It brings together multiple published methods and freely available software to process Hyperion images, and includes algorithms adapted to the batch processing of large quantities of images. Its outputs have been shown to be similar to those of a commercially available software largely used by the scientific community to process Hyperion data. Apart from the fact that it is freely distributed, one of the main advantages of the package is that it is open source and easily tunable to the specific needs of each user. Further works should focus on implementing a cloud and cloud shadows detection, improving the haze removal method, and accelerating the rough terrain atmospheric correction.

## Figures and Tables

**Table 1:** Median absolute and relative reflectance differences between SUREHYP and FLAASH outputs over the 400–2500 nm range for area (d) (see Figure 6 for the location).

	Blue	Green	Red	NIR	SWIR
Spectral Range (nm)	450–510	510–580	580–750	750–1000	1000–2500
absolute diff. [0–1]	−0.008	−0.032	−0.020	0.022	0.006
relative diff. (%)	−4.4	−14.6	−9.7	10.7	3.76

## Data Availability

Not applicable.
